# Oral supplementation with fish cartilage hydrolysate in an adult population suffering from knee pain and function discomfort: results from an innovative approach combining an exploratory clinical study and an ex vivo clinical investigation

**DOI:** 10.1186/s12891-023-06800-4

**Published:** 2023-09-21

**Authors:** Henrotin Yves, Julie Herman, Melanie Uebelhoer, Fabien Wauquier, Line Boutin-Wittrant, Anne-Françoise Donneau, Justine Monseur, Variance Mokam Fotso, Marie Duquenne, Mélanie Wagner, Elodie Bouvret, Bérénice Costes, Yohann Wittrant

**Affiliations:** 1 Service de Kinésithérapie et de Réadaptation Fonctionnelle, Hôpital Princesse Paola, Vivalia, Rue du Vivier 21, Marche-en-Famenne, 6900 Belgium; 2 musculoSKeletal Innovative research Lab (mSKIL), University of Liège, Liège, 4000 Belgium; 3 Artialis SA, Avenue de l’Hôpital 11, Liège, 4000 Belgium; 4 Clinic’n’Cell SAS, UFR de Médecine et de Pharmacie, 28 Place Henri Dunant, Clermont-Ferrand, TSA 50400 63001 France; 5 Département des Sciences de la Santé publique, UR BSTAT ULiège, Université de Liège, Avenue Hippocrate 13, Liège, 4000 Belgium; 6 Centre Médical Chant d’oiseau, Avenue des Frères Legrain 85, Woluwe-Saint Pierre, 1150 Belgium; 7 Abyss Ingredients, 860 Route de Caudan, Caudan, 56850 France; 8 INRAE, UNH, Clermont-Ferrand, 63001 France; 9 Université Clermont Auvergne, UFR de Médecine de de Pharmacie, 28 Place Henri Dunant, Clermont-Ferrand, TSA 50400 63001 France

**Keywords:** Chondroitin sulfate, Fish cartilage hydrolysate, Joint discomfort, Pain, Clinical efficacy, Nutraceuticals

## Abstract

**Background:**

Aging is frequently associated with impairments of the musculoskeletal system and many elderly people experience joint discomfort or pain which might reduce their ability to move and consequently alter their quality of life. A beneficial effect of fish cartilage hydrolysate (FCH) on pain and joint function has recently been shown in an ACLT/pMMx osteoarthritis rat model.

**Methods:**

We therefore performed an exploratory, non-comparative, multi-centric clinical trial including 33 subjects with moderate knee joint discomfort and loss of functionality to investigate the efficacy of FCH on their algo-functional status. We further determined the potential health benefit of FCH in an original clinical ex vivo study investigating the role of FCH human metabolites on primary human chondrocytes.

**Results:**

FCH significantly improved knee pain and function, as assessed by the Knee injury and Osteoarthritis Outcome Score (KOOS). Moreover, FCH significantly reduced pain at rest and while walking, and patient global assessment (PGA), as assessed by the Visual Analogue Scale (VAS), and improved patients’ quality of life (SF-36). FCH metabolites decreased the synthesis of catabolic factors (MMP-13) and pro-inflammatory mediators (NO, PGE2) and limited the inhibitory effect of IL-1β on the synthesis of cartilage matrix components (GAG and collagen).

**Conclusions:**

Thus, these data provide insights on the mode of action of FCH in humans and contribute to explain how FCH may relieve pain and improve joint function in subjects with knee discomfort. Although these preliminary data need to be confirmed in a randomized controlled trial, they strongly support the potential health benefit of such an active ingredient. **Trial registration**: The study was registered on clinicaltrials.gov with the identifier NCT04420091 (09/06/2020).

**Supplementary Information:**

The online version contains supplementary material available at 10.1186/s12891-023-06800-4.

## Background

Modern Western societies are facing the challenges of an ever-aging population, where life expectancy and the proportion of older people in the population continue to increase. According to the United Nations, at least 1.5 billion people aged 65 years or older are expected for 2050, compared to 703 million in 2019 [1]. Living longer comes with an increased risk of age-related ailments and diseases, and thus the necessity of effective preventive and therapeutic measures.

Aging is frequently associated with impairments of the musculoskeletal system (joints, bones, and muscles), and many elderly people experience joint discomfort or pain which might reduce their ability to move [2]. Mobility is a core indicator of health and function in aging, and its impairment greatly affects patients’ quality of life (QoL).

Current medical treatments of joint discomfort are mainly symptoms-oriented and rely on analgesic and nonsteroidal anti-inflammatory drugs (NSAIDs) to relieve pain and inflammation [3–6]. Chronic use of these drugs can however lead to severe adverse effects such as hepatotoxicity, loss of kidney function, or gastrointestinal bleeding [7–10].

As an alternative, high-quality food supplements are being explored for the prevention and management of joint discomfort [11]. As evidenced by various randomized clinical trials, certain natural compounds (i.e., avocado/soybean unsaponifiable extract, *B. serrata* extract, pycnogenol, *C. longa* extract, and passion fruit peel extract) may lead to clinically important short-term improvement of pain and function in patients with hand, hip, or knee Osteoarthritis (OA) [12].

Glucosamine sulfate (GS) and chondroitin sulfate (CS) are important basic natural components of cartilage. They are naturally formed by the body but can also be provided in the diet. Supplementation may be especially beneficial when there is a disturbed balance between catabolic and anabolic processes. While several in vitro and in vivo experiments have shown that these compounds taken alone or in combination can modify or stabilize joint disorders, conflicting and controversial results have been observed in clinical trials with regards to their symptomatic and structure-modifying effect [13–21]. Additional human studies would therefore be helpful to establish their clinical efficacy.

A dried and concentrated fish cartilage hydrolysate (FCH) including the glycosaminoglycans GS and CS together with collagen hydrolysate to support joint cartilage has recently been demonstrated to have beneficial effects on pain and joint function in an in vivo efficacy study [22]. This product has been tested in an exploratory clinical study aiming to determine whether its oral administration can contribute to the improvement of mobility and joint function in an adult population with knee discomfort and in an *ex vivo* study investigating the biological activity of serum from subjects who have orally ingested FCH on cultures of human primary chondrocytes.

## Materials and methods

### Study design and selection of subjects of the exploratory clinical study

This study was an exploratory, non-comparative, multi-centric trial in a minimum of 30 free-living healthy male and female subjects with moderate knee joint discomfort and loss of functionality. It included subjects from 2 centers in Belgium who were enrolled in the study from June 11th, 2020, to December 17th, 2020. The main inclusion criteria were age between 40 and 80 years with a body mass index (BMI) ≤ 35 kg/m^2^, a knee discomfort score at rest over the last month on the most painful knee evaluated on VAS ≥ 40 mm and being able to follow the instructions of the study. The main exclusion criteria were recent trauma (< 1 month) of or prosthesis in the symptomatic knee, concurrent articular disease interfering with the evaluation of knee pain, corticosteroid injection in the target knee in the last month, hyaluronan injection in the target knee, or arthroscopy in the last 6 months, oral corticotherapy in the last 3 months, symptomatic slow-acting drugs for osteoarthritis (SYSADOA) or dietary supplements used for joint health in the last 3 months, allergy or contraindication to the product components, or treatments based on strontium ralenate, bisphosphonates, selective estrogen-receptor modulator (SERM) and parathormone (PTH) in the last 12 months. A detailed list of exclusion criteria can be found in Supplemental Table S12. Subjects were allowed to take rescue medication to manage knee pain (i.e., paracetamol and non-steroidal anti-inflammatory drugs (NSAIDs)) but were asked to stop 24 hours before each visit.

All subjects enrolled in the study were taking 2 capsules of FCH 500 mg each, once daily in the morning, preferably during a meal, with water, for 3 months. The FCH (Cartidyss® NG, Abyss Ingredients, Caudan, France) is a powder obtained from a standardized manufacturing process based on enzymatic hydrolysis of fish cartilage without preservatives or processing aids. Its natural composition contains collagen peptides (< 3000 Da), chondroitin sulfate and minerals, respectively 65%, 26%, and 9%, given as an indicative value. Written informed consent to participate in the study was obtained from all subjects before enrolment.

### Outcome measures

The primary objective was to evaluate the effect of FCH on knee pain and function after 3 months of supplementation using the self-administered questionnaire KOOS. The secondary objectives were to evaluate the effects of FCH on knee pain and function using KOOS, knee pain at rest or while walking, PGA using VAS, QoL using SF-36, and the responder rate based on the Osteoarthritis Research Society International - Outcome Measures in Rheumatoid Arthritis Clinical Trials (OARSI-OMERACT) criteria after 1 month and 3 months. Product satisfaction and tolerance by self-administered questionnaires, as well as subjects’ compliance based on tablet counts and consumption of pain killers, were also assessed.

### Study design and selection of the ex vivo study

The study was conducted in accordance with the Declaration of Helsinki of 1975 (https://www.wma.net/what-we-do/medical-ethics/declaration-of-helsinki) revised in 2013. The human study was approved by the French Ethical Committee (2021-ND77 RIPH2 HPS / N° SI RIPH: 21.01436.000014 / N° EudraCT/ID RCB: 2021-A01773-38 / Comité de Protection des Personnes CPP Paris, Ile-de-France 1; approved 08 October 2021). The volunteers were informed of the objectives and the potential risks of the present study and provided their written informed consent before they participated in the study.

Relevance of such a clinical *ex vivo* approach was originally validated by Wittrant’s group [23–27]. The protocol design for this trial was very recently published by Wauquier et al. [28]. Briefly, a pool of 10 healthy men (age: 25.4 years old, +/−3.7; BMI: 23.6 kg/m^2^, +/−1.9; >60 kg; without drug treatment; and no distinction on ethnicity) volunteered for this study. Their enrolment was validated upon consistent blood formulation, renal (urea and creatinine) and liver functions (aspartate aminotransferase (AST), alanine aminotransferase (ALT), gamma-glutamyltransferase (GGT) activities). Then, volunteers fasted for 12 h before they were given 12 g of FCH. The dose was set according to validated preclinical [29] and clinical data [24, 30–33]. According to the hydroxyproline absorption profile determined during the pharmacokinetic phase of the study, volunteers were subjected to blood sampling before FCH administration (naïve serum) and after treatment with FCH (enriched serum). Approximately 48 mL of venous blood was drawn from the cubital vein before the ingestion for the collection of a naïve serum. At the maximum absorption peak, 48 mL of blood was drawn for enriched serum collection. Serum preparation and collection were performed at the Centre d’Investigation Clinique de Clermont-Ferrand—Inserm 1405, a dedicated research department that ensures the quality of samples and the compliance with regulatory and ethical laws (French standard certification NF-S-96900). Serum was stored at − 80°C until analysis.

### Human primary chondrocyte cultures

Human articular chondrocytes (HACs) were harvested from tibial plateau and femoral condyles following knee replacement surgery and isolated as previously described previously [25]. Only intact cartilage areas were kept and processed for chondrocytes isolation. Briefly, cartilage was sliced and chips were successively digested at 37°C with 0.05% type IV-S hyaluronidase (750–3000 units/mg) (Sigma-Aldrich,Lyon, France) in Hank’s Balanced sodium Salt (HBSS) (Life Technologies, Villebon-Sur-Yvette, France) for 10 min and then with 0.2% trypsin (≥ 9000 BAEE units/mg) (Sigma-Aldrich, Lyon, France) for 15 min and with 0.2% type II collagenase (125 units/mg) (Sigma-Aldrich, Lyon, France) for 30 min. Cartilage chips were then digested overnight at 37°C in 0.03% type II collagenase in control medium (DMEM supplemented with 10% Fetal calf serum (FCS) (Pan-Biotech, Aidenbach, Germany) and 1% penicillin/streptomycin (P/S; Life Technologies, Villebon-Sur-Yvette, France) [25]. Cells were plated at passage 1 in F225 flasks at a density of 100,000 cells/cm² and maintained at 37°C in a humidified atmosphere of 5% CO2 in control medium (10% FCS, 1% P/S). At confluency, cells were subcultured for *ex vivo* experiments. To analyze the effects of FCH-enriched serum metabolites, cells were preincubated for 24 h in DMEM in the presence of 10% of human-enriched serum according to the Clinic’n’Cell protocol (DIRV INRA 18–00058) (1% P/S) prior an additional 24 h treatment with human recombinant IL-1β (Millipore Corporation, Molsheim, France) at 1 ng/mL.

### Cell viability

The *ex vivo* cell viability was determined using an XTT-based method (Cell Proliferation Kit II, Sigma-Aldrich) according to the supplier’s recommendations. Optical density was measured at 450 nm.

### NO, PGE2, MMP-13, collagen II and aggrecan quantification

Nitrate/Nitrite colorimetric assay and prostaglandin E2 Enzyme Immunoassay (EIA) kits were obtained from Cayman Chemical (Ann Arbor-MI, USA). Human ELISA Kits for MMP-13, Collagen II and Aggrecan detection were purchased from Abcam® (Paris, France). The NO, PGE2, MMP-13 and Aggrecan level measurements were performed according to manufacturer’s instructions in cell supernatants whereas collagen II amount was measured in cell lysate. For human serum, measurements were performed in quadruplicates for each sample of the ten volunteers.

### GAG assay

A dimethylmethylene blue (DMB) assay was used to detect GAG production in cell lysates as previously described [34]. DMB solution was prepared at final concentration of 46 mmol/L in a pH 3 adjusted buffer: 40 mmol/L NaCl, 40 mmol/L glycine. Sample concentrations were determined by mixing 50 µL of cell extract with 200 µL of DMB reagent. Following 30 min incubation, the absorbance was read at 595 nm on an EL_X_808 IU spectrophotometer (BioTek Instruments, USA). The GAG content was determined using a standard curve of chondroitin sulfate (Sigma). Results are expressed as µg of GAG per mg of total proteins determined by BCA assay (Sigma).

### Cell lysis

Cells were lysed using lysis buffer (50 mmol/L Tris pH 7.8, 150 mmol/L NaCl, 0.5% sodium deoxycholate, 1% NP40), and each fraction was stored at − 80℃ until analyses.

### Protein quantification

Protein contents were determined by the BCA Protein Assay Kit (Millipore 71285-M). The BCA protein assay is based on a biuret reaction, which is the reduction of Cu2 + to Cu + by proteins in an alkaline solution with concentration-dependent detection of the monovalent copper ions. Bicinchoninic acid is a chromogenic reagent that chelates the reduced copper, producing a purple complex with strong absorbance at 562 nm.

### Statistical analysis

For the exploratory clinical study, statistical analysis was performed on the full analysis set (FAS) population following the guidelines on statistical principles for clinical trials (ICH E9). Due to the exploratory nature of the study, the number of subjects to be recruited has been estimated based on a potential “medium” effect size of 0.60, which is more than the commonly observed effect of the placebo in clinical trials on articular disorders in the knee (0.50) [35]. With a two-tailed t-test on two dependent means, a power of 80%, and an alpha error of 0.05, the required sample size for an effect size of 0.60 was estimated to be 24 with the software GPower. To compensate for dropouts, the sample size was inflated by 20%, and a total of 33 subjects were recruited for the study.

Quantitative variables were summarized with classical descriptive statistics (n, arithmetic mean, standard deviation, median, interquartile range) at each time point. Counts and percentages were reported at each time for qualitative variables. Median (P25-P75) was also reported for ordinal variables. Assessment of the normality of the distribution of the quantitative variable was investigated numerically by comparing mean and median and graphically using histogram and quantile-quantile plot. Shapiro-Wilk normality test completed the assessment of the normality. In results tables, quantitative variables with a normal distribution were expressed as mean (± SD). Median (Q1-Q3) was used when normality was not fulfilled.

The primary target variable, KOOS global score, was analyzed using a Student t-test for paired samples. The time evolution of the following quantitative variables was analyzed using repeated-measures ANOVA model when normality distribution was fulfilled, and Friedman’s test for variables with dissymmetric distribution:

- The global score of the KOOS and its five subscales: Pain, Symptom, Activity of Daily Living, Sport and Recreation function and Quality of Life (the global score was calculated by summing up the score of all five subscales);
- Each subscale of the 36-item Short-Form Health Survey (SF36) and the global score of quality of life obtained by summing up the score of each subscale;
- Knee pain at rest and while walking using the Visual Analog Scale (VAS);
- VAS for PGA.

When appropriate, a natural logarithmic transformation was performed to achieve residuals normality assumption. Multiple pairwise comparisons between time points were performed in case of significant time evolution. Effect size between baseline and the end time point was calculated for KOOS global score and subscales, for knee pain at rest and while walking, and for PGA. Effect size calculated as Cohen’s d was obtained by dividing the mean difference by its standard deviation. For effect size associated to knee pain at rest and while walking and for effect size associated to PGA, the absolute value of the mean difference was considered.

Compliance and subjects’ satisfaction were compared between the first and the second follow-up using Wilcoxon’s sum of rank test. The rate of supplementation responder was compared between the first and the second follow-up with McNemar’s test. Time evolution of the use of pain killers for knee pain (yes/no) was analyzed using a generalized linear model for repeated measures. Time evolution of the number of days of rescue treatments uptake was modeled using a Friedman’s test.

Statistical significance was achieved at 95% confidence (p-value significance < 0.05). Due to the exploratory nature of the study, no multiplicity adjustment was implemented.

For the *ex vivo* study, prism V.9.4.1 (GraphPad Software) was used to run statistical tests and draw figures. The following statistical plan was applied: A Shapiro–Wilk normality test was used to determine whether the data are consistent with a Gaussian distribution. If data were not distributed according to the normal distribution, a Kruskal–Wallis nonparametric test was used followed by Dunn test for post hoc for multiple comparisons or a Mann-Whitney test for comparison between 2 groups. When normal distribution and equal variance was assumed, measures were subjected to one-way ANOVA with Tukey’s test for multiple comparisons. Values are presented as the means ± SEM unless specified otherwise. The differences were considered statistically significant at p < 0.05 with * for p < 0.05; ** for p < 0.01; *** for p < 0.001; **** for p < 0.0001 and ns for p > 0.05.

## Results

### Exploratory clinical study

#### Demographics and other baseline characteristics

**Fig. 1:**
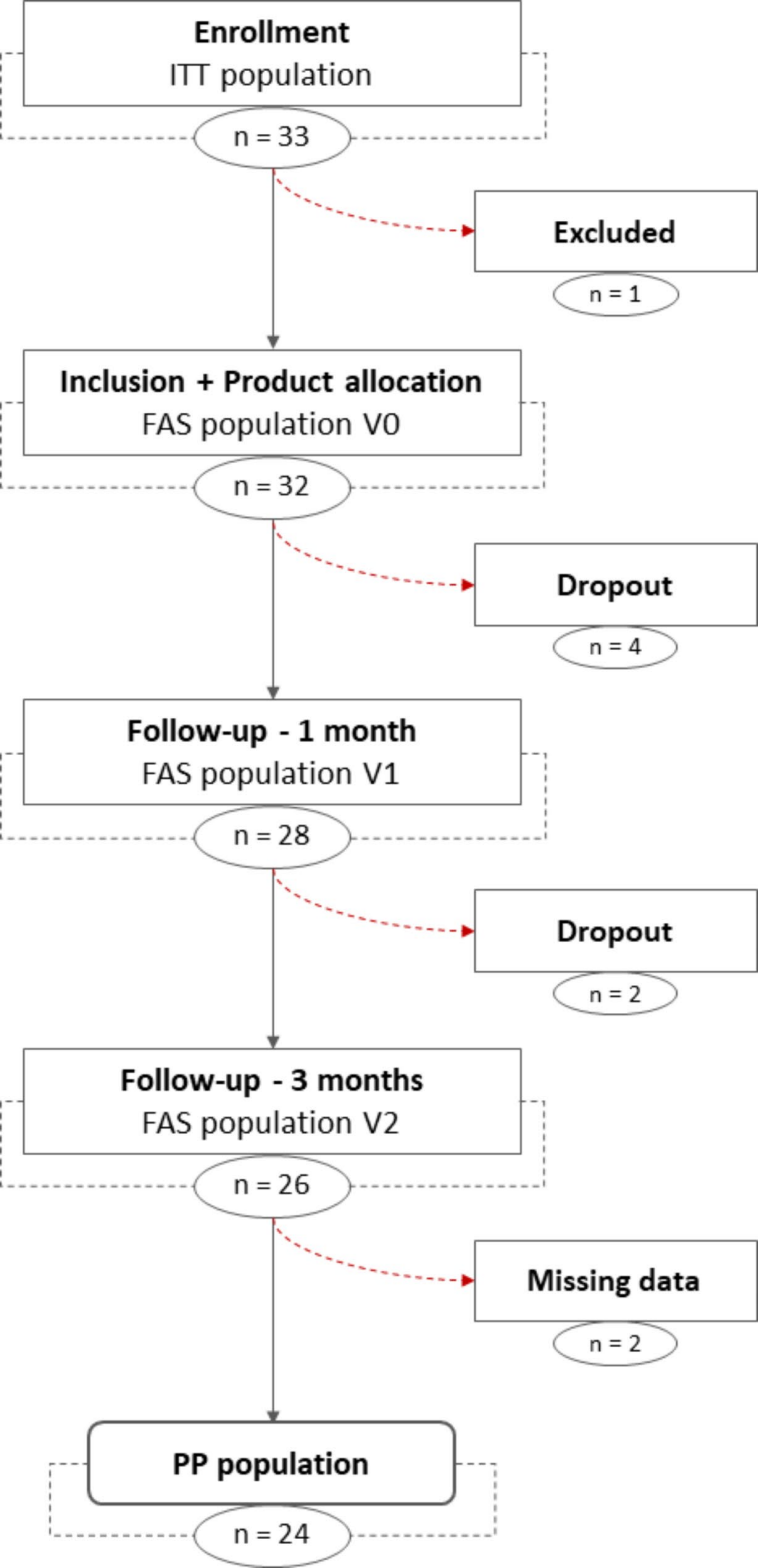
Flow-chart of subject disposition. ITT, intention to treat; FAS, full analysis set; PP, per protocol. Safety population n = 32

Out of the pre-screened subjects, 33 were included in the intention-to-treat (ITT) group. A total of 32 subjects were included in the study without any eligibility violation and took at least one dose of the product, constituting the FAS population. The per-protocol (PP) population included 24 subjects of the FAS who completed the study and did not have any major protocol deviation (Fig. 1). While results for PP and FAS were very much the same, only those of the FAS are included in the main text, while efficacy outcomes for PP can be found in the supplementary data. The general characteristics of the FAS at baseline are summarized in Table 1. Subjects were mainly male (65.6%), Caucasian (96.9%), and aged 64.2 ± 8.6 years. Their mean body mass index was 26.9 ± 3.3 kg/m².

**Table 1:** Demographic characteristics of the subjects – FAS population

Variable	N	Category	N(%)	Mean	SD	Min	Q1	Median	Q3	Max	
Age (year)	32			64.19	8.61	42.00	59.50	65.50	70.00	78.00	
Sex	32										
		Male	21 (65.6)								
		Female	11 (34.4)								
Height (cm)	32			174.69	8.70	156.00	168.00	175.00	182.00	190.00	
Weight (kg)	32			81.98	11.85	54.00	75.00	80.50	86.50	107.00	
BMI (kg/m²)	32			26.86	3.31	20.50	24.67	26.81	29.08	33.30	
Ethnic	32										
		Caucasian	31 (96.9)								
		Other	1 (3.1)								

Among the 32 subjects included in the FAS population at baseline, the mean score of the Visual Analogue Scale (VAS) for knee pain at rest was 53.66 ± 11.95, and the mean score of the VAS for knee pain while walking was 61.56 ± 21.30. The mean for the Patient Global Assessment (PGA) scale was 64.3 ± 12.9 mm (Table S1).

#### KOOS global score and subscales – pain and function

**Fig. 2:**
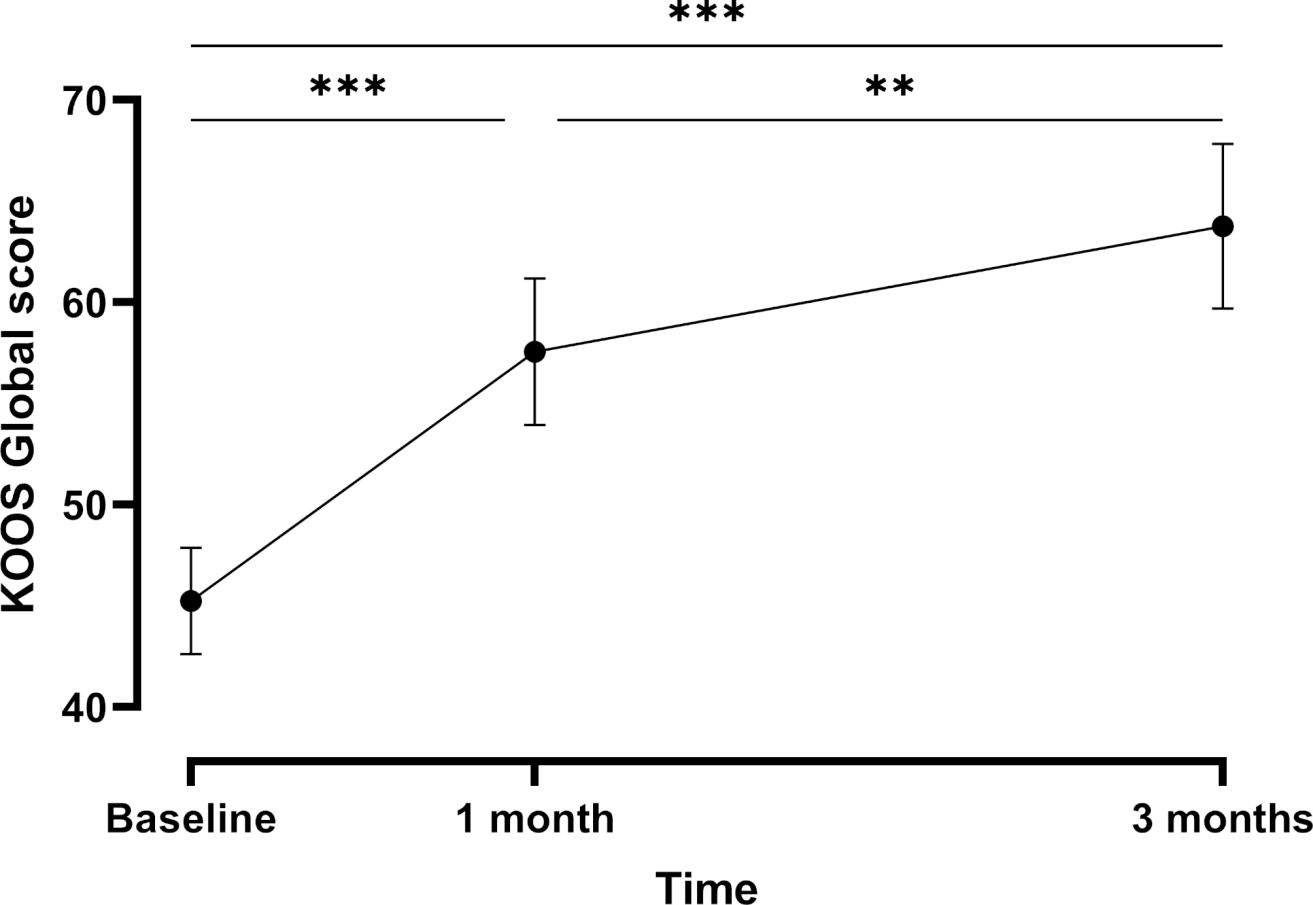
Mean evolution (± SE) of the KOOS global score over time – FAS population. ***p ≤ 0.001; **p ≤ 0.01

Global Knee injury and Osteoarthritis Outcome Score (KOOS) and its five subscales concerning pain, symptoms, activity of daily living, sport and recreation function, and quality of life increased significantly over time (Table 2, Figs. 2 + S1), meaning an improvement of the subject’s algo-functional status. They were significantly higher at 1 month and 3 months compared to baseline. KOOS global score and a subset of its subscales (KOOS pain score, KOOS symptoms score, KOOS score about daily living activities, and KOOS score concerning the quality of life) were also greater after 3 months than after 1 month of follow-up. Interestingly, effect size between baseline and 3 months of follow-up indicated a large effect for KOOS global score and all subscales (Table 2). The results for the PP population were similar and are summarized in supplementary table S2.

**Table 2:** Results of the repeated measures ANOVA models for KOOS global score and each subscale, and mean difference and effect size between baseline and 3 months of follow-up – FAS population

Variable	Baseline (n = 32)	After 1 month (n = 28)	After 3 months (n = 26)	P-value	Mean difference (n = 26)	Effect size
Global score [mean ± SD]	45.24 ± 14.93	57.56 ± 19.09	63.76 ± 20.76	*< 0.0001*	19.31 ± 16.14	1.20
Pain* [mean ± SD]	48.26 ± 16.32	61.11 ± 19.74	70.09 ± 19.44	*< 0.0001*	22.44 ± 17.91	1.25
Symptom [mean ± SD]	55.58 ± 20.48	68.37 ± 20.01	73.76 ± 21.26	*< 0.0001*	18.54 ± 20.40	0.91
Activity of daily living [mean ± SD]	54.37 ± 16.96	65.23 ± 20.97	72.23 ± 20.83	*< 0.0001*	18.21 ± 13.24	1.38
Sport and recreation function [mean ± SD]	25.63 ± 16.40	40.18 ± 25.00	43.85 ± 28.86	*< 0.0001*	18.85 ± 22.46	0.84
Quality of life* [mean ± SD]	42.38 ± 19.10	52.90 ± 20.94	58.89 ± 24.95	*< 0.0001*	18.51 ± 20.35	0.91

*log-transformed variable

#### SF-36 global score and subscales – quality of life

**Fig. 3:**
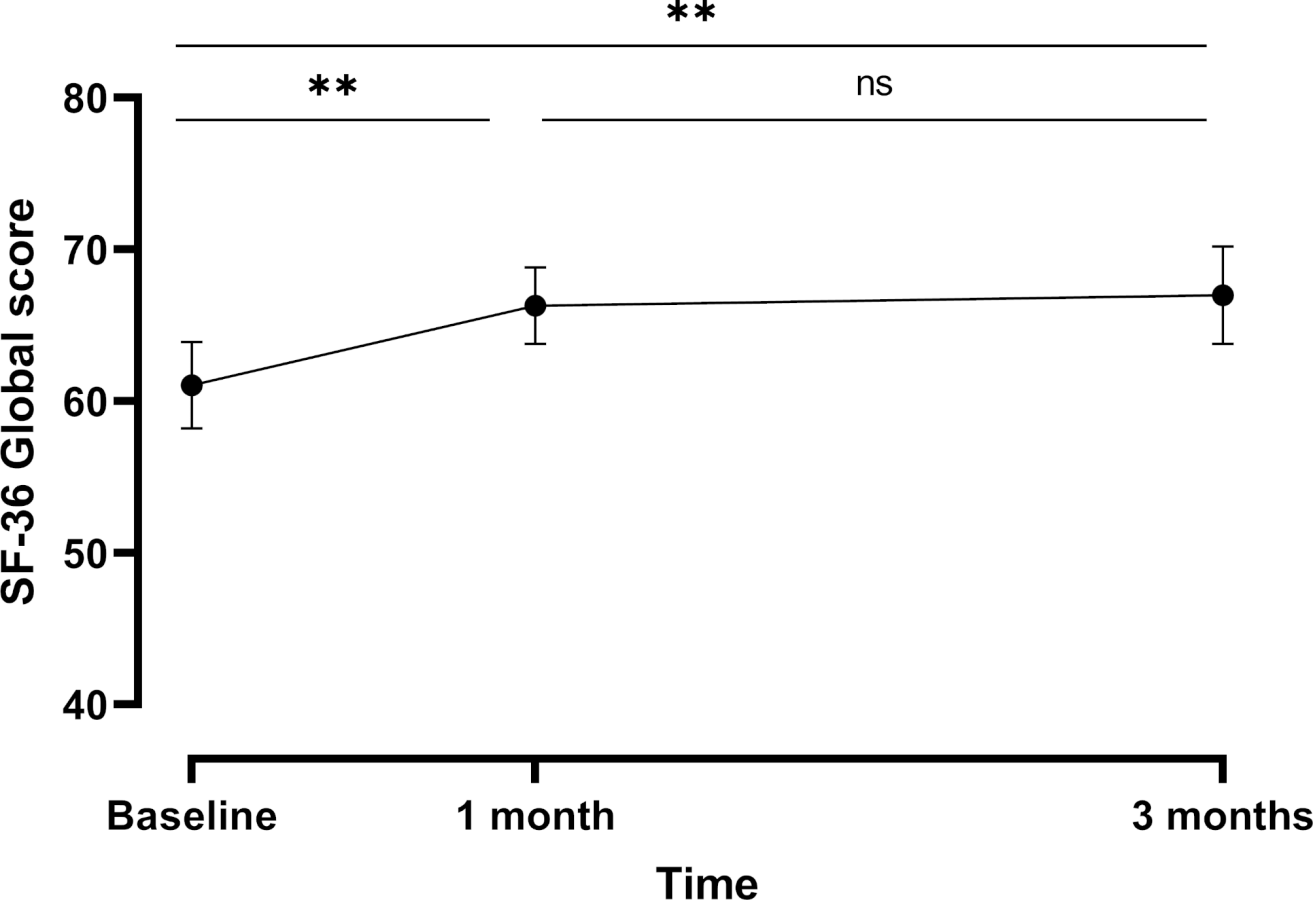
Mean evolution (± SE) of the SF-36 global score over time – FAS population. **p ≤ 0.01; ns, not significant

The Short Form [36] Health Survey (SF-36) global score and its subscales concerning physical functioning, role limitation due to physical health, energy and fatigue, pain, and general health significantly improved over time. Subscales about role limitation due to emotional problems, emotional well-being, and social functioning, on the other hand, were not significantly impacted over time (Table 3 and Fig. 3 + S2). The results for the PP population were similar and are summarized in supplementary table S3.

**Table 3:** Results of the repeated measures ANOVA models for SF-36 global score and each subscale – FAS population

Variable	Baseline (n = 32)	After 1 month (n = 28)	After 3 months (n = 26)	P-value
Global score [mean ± SD]	61.05 ± 16.09	66.30 ± 13.93	66.99 ± 16.34	*0.0024*
Physical functioning [mean ± SD]	56.72 ± 21.43	58.21 ± 20.69	65.77 ± 23.52	*0.0009*
Role limitations due to physical health [mean ± SD]	52.34 ± 39.84	76.79 ± 31.13	75.96 ± 36.39	*0.0027*
Role limitation due to emotional problems [median (Q1-Q3)]	100.00 (66.67–100.00)	100.00 (66.67–100.00)	100.00 (33.33–100.00)	0.9406*
Energy/fatigue [mean ± SD]	55.78 ± 14.49	64.29 ± 15.01	55.96 ± 18.55	*0.0030*
Emotional well-being [mean ± SD]	65.25 ± 19.71	67.71 ± 17.52	62.77 ± 19.49	0.1660
Social functioning [median (Q1-Q3)]	75.00 (56.25–93.75)	81.25 (62.50–100.00)	75.00 (62.50–100.00)	0.5302*
Pain [mean ± SD]	50.86 ± 19.21	61.61 ± 15.96	64.90 ± 23.37	*< 0.0001*
General health [mean ± SD]	67.50 ± 12.95	64.82 ± 12.87	71.54 ± 11.47	*0.0223*

* Friedman’s test p-value

#### VAS – pain and function

**Fig. 4:**
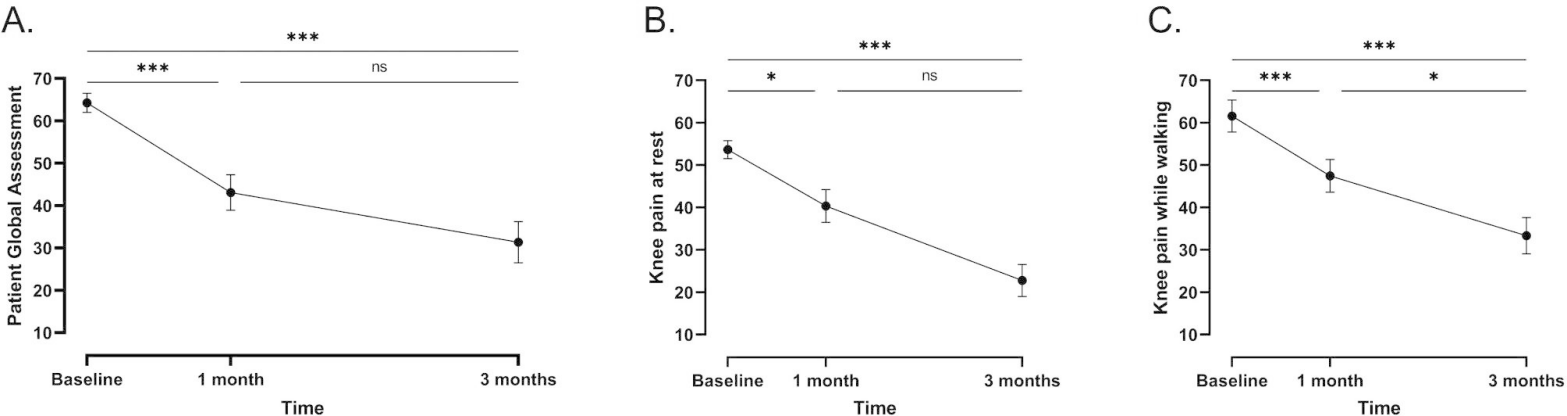
Mean evolution (± SE) of (**a**) PGA using VAS scale over time; (**b**) Knee pain at rest over time; (**c**) Knee pain while walking over time - FAS population. ***p ≤ 0.001; *p ≤ 0.05; ns, not significant

In comparison to baseline, VAS knee pain at rest and while walking decreased significantly after 1 month and 3 months of follow-up (Table 4 and Fig. 4). In addition, a significant reduction was observed for knee pain while walking between the two follow-up visits, but not for knee pain at rest. PGA decreased significantly between baseline and the 1-month and 3-month visits respectively. The effect size between baseline and the second follow-up indicated a large effect for all three variables (Table 4). The results for the PP population were similar and are summarized in supplementary table S4.

**Table 4:** Results of the repeated measures ANOVA models for knee pain at rest and while walking and PGA using VAS scale, and mean difference and effect size between baseline and 3 months of follow-up – FAS population

Variable	Baseline (n = 32)	After 1 month (n = 28)	After 3 months (n = 26)	P-value	Mean difference (n = 26)	Effect size
At rest* (mm) [mean ± SD]	53.66 ± 11.95	40.38 ± 20.50	22.77 ± 19.34	*0.0005*	-30.85 ± 21.14	1.46
While walking (mm) [mean ± SD]	61.56 ± 21.30	47.45 ± 20.42	33.35 ± 21.68	*< 0.0001*	-29.38 ± 29.82	0.99
PGA (mm) [mean ± SD]	64.28 ± 12.90	43.10 ± 22.24	31.38 ± 24.74	*< 0.0001*	-33.42 ± 31.02	1.08

*log-transformed variable

#### Responder rate

The ratio of OARSI-OMERACT responders in the FAS population was 46.1% after 1 month and 65.4% after 3 months of FCH supplementation. This difference was however not statistically significant (Table 5). Out of 14 non-responder 1 month of supplementation, 7 stayed non-responders while 7 became responders after 3 months; out of 12 responders after 1 month, 2 became non-responders and 10 stayed responders (Table 5). The results for the PP population were similar and are summarized in supplementary table S5.

**Table 5:** Comparison of subjects’ supplementation response between the first and the second follow-up – FAS population

		After 3 months [N (%)]			
		No	Yes	Total	P-value
After 1 month [N (%)]	No	7 (26.9)	7 (26.9)	14 (53.9)	0.0956
	Yes	2 (7.7)	10 (38.5)	12 (46.1)	
	Total	9 (34.6)	17 (65.4)	26 (100.0)	

#### Compliance, subjects’ satisfaction, and pain killer use

Compliance was high at each visit and did not significantly differ after 1 month or 3 months of follow-up (97.65–100.00% vs. 95.24–100.00% respectively) (Table S6). On a scale from 1 to 5 (1 = extremely unsatisfied; 2 = unsatisfied; 3 = neither satisfied nor unsatisfied; 4 = satisfied; 5 = extremely satisfied), subjects were satisfied regarding taste, oral intake, digestion and effects on symptoms and on quality of life (Table S7). Pain killer consumption did not change over time. No increase was observed in neither the use nor the frequency of pain killer intake (Table S8).

#### Adverse events

Only one Serious Adverse Event (SAE) (a heart attack) was reported during the study, but it was unrelated to the FCH. Only a few minor Adverse Events (AE) (i.e., gastro-intestinal, urinary, or skin disorders, see Table S9 for a complete list of AEs and SAEs) were probably or definitively related to the FCH (Table 6). In total, four subjects suspended or stopped the product due to an AE (Table S10) amongst the six subjects who dropped out of the study. The number of drop-outs increased with time and was around 20% after 3 months (Table S11).

**Table 6:** Distribution of the link of AE and SAE with FCH– Safety population – N = 28 adverse events

	N (%)
AE link with FCH	
Unrelated	1 (3.6)
Unlikely	18 (64.3)
Probable	5 (17.9)
Related	4 (14.3)
SAE link with FCH	
Unrelated	1 (100.0)

Taken together, our data suggest an improvement of the articular function. In order to further demonstrate the biological activity of FCH on cartilage tissue and provide clues on the mechanisms likely contributing to its benefits for human articular function, we investigated the influence of human circulating metabolites following FCH ingestion on primary human articular chondrocytes behavior *ex vivo*.

### Pharmacokinetic and ex vivo study

#### Kinetic Profile of FCH absorption

**Fig. 5:**
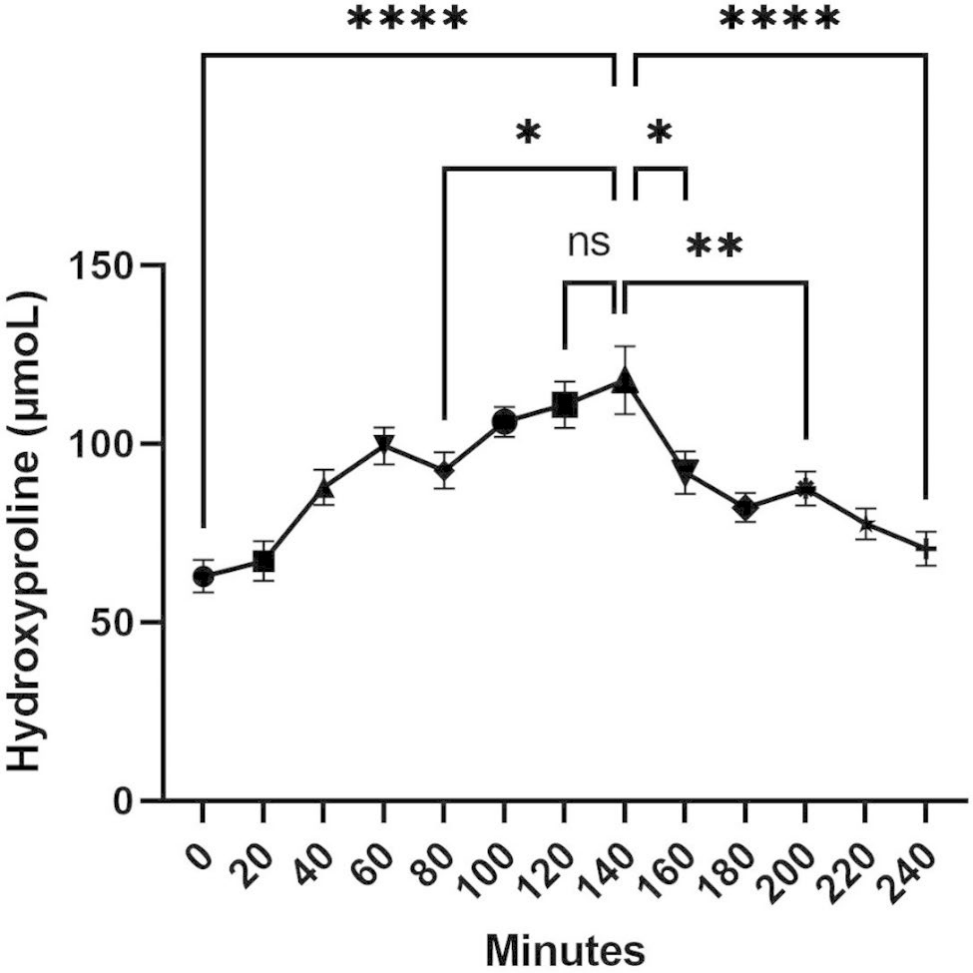
Evolution of the concentration of circulating hydroxyproline in blood. Values are presented as mean ± SEM. The differences were considered statistically significant at p < 0.05 with * for p < 0.05; ** for p < 0.01; *** for p < 0.001; **** for p < 0.0001 and ns for p > 0.05 [28]

Briefly, fasted volunteers received 12 g of FCH, and the absorption profile was monitored during a 240 min time period by measuring hydroxyproline blood levels. Analyses showed that following ingestion of FCH, circulating concentration of hydroxyproline continuously increases to reach a maximum of 117.7 µM at 140 min post ingestion (+ 87.5% compared to basal level), before returning to almost basal level by the end of the kinetics (Fig. 5), thus supporting that FCH is well absorbed and available. This has been previously described and published by Wauquier et al. [28].

Based on these results, both naïve and enriched serum with FCH metabolites for the ex vivo cultures were subsequently collected before ingestion (naïve serum) and at 140 min post-ingestion (enriched serum).

#### FCH human metabolites limit unbalanced metabolism upon inflammatory stress

To ensure the physiological relevance of our *ex vivo* approach, we checked that neither naive nor enriched human sera processed according to the Clinic’n’Cell methodology (DIRV#18–0058; see the Patents section) impaired cell growth (Figure S3).

**Fig. 6:**
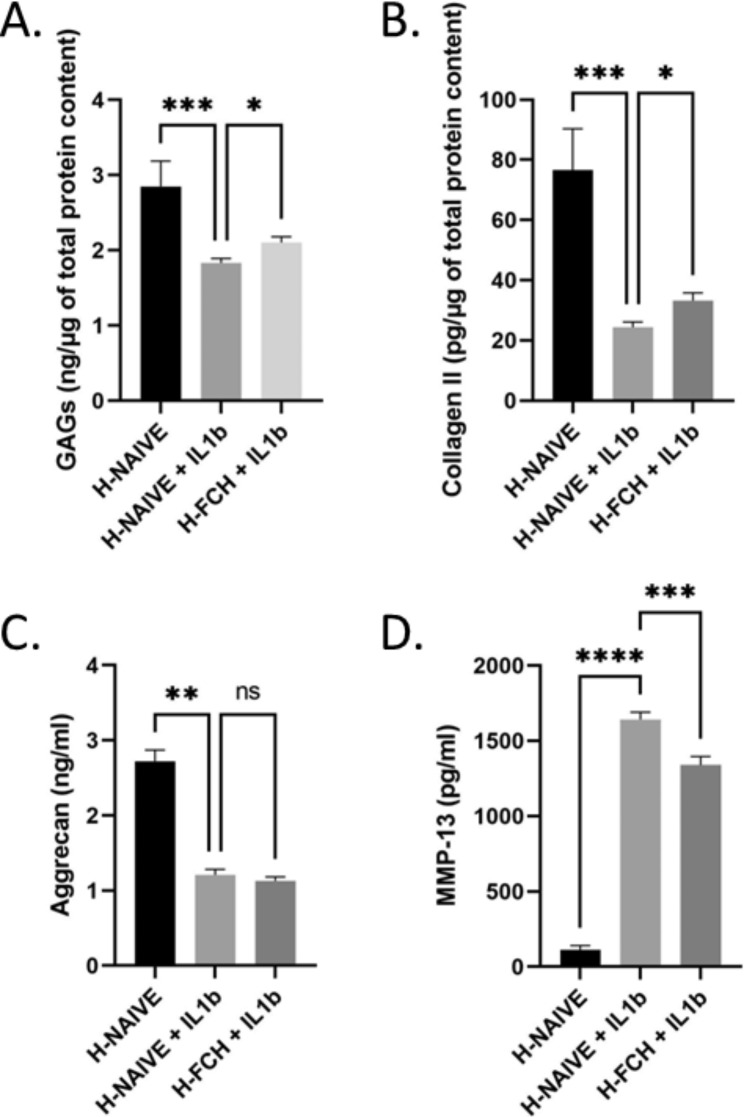
Primary human chondrocytes were incubated in the presence or absence of rhIL-1b (1ng/ml) in combination with either human naive serum (H-NAIVE) or human serum enriched with metabolites derived from FCH ingestion (H-FCH). Glycosaminoglycans (GAGs) (**A**), Collagen II (**B**), Aggrecan (**C**) and MMP-13 (**D**) levels. Measures were performed in quadruplicates per condition/volunteer (n = 10 volunteers). Values are presented as mean ± SEM. The differences were considered statistically significant at p < 0.05 with * for p < 0.05; ** for p < 0.01; *** for p < 0.001; **** for p < 0.0001 and ns for p > 0.05

As expected and shown in Fig. 6, when cultured with IL-1β, primary human articular chondrocytes showed both decreased GAG and collagen II protein content with a reduction of -35% and − 68% respectively compared to non-stimulated controls. Along, IL-1β stimulated MMP-13 protein level by + 1382%. The presence of human metabolites derived from FCH absorption slightly but significantly limited IL-1 β related effects. Reduction of both GAGs and collagen II were constrained to -26% and − 56% respectively, and IL-1 β -induced MMP-13 production was limited to + 1107% in the presence of metabolites. In contrast, while IL-1β decreased aggrecan protein content by -55%, no significant effect of FCH metabolites was observed on this parameter.

#### FCH human metabolites temper the production of secondary inflammatory mediators by human primary chondrocytes

**Fig. 7:**
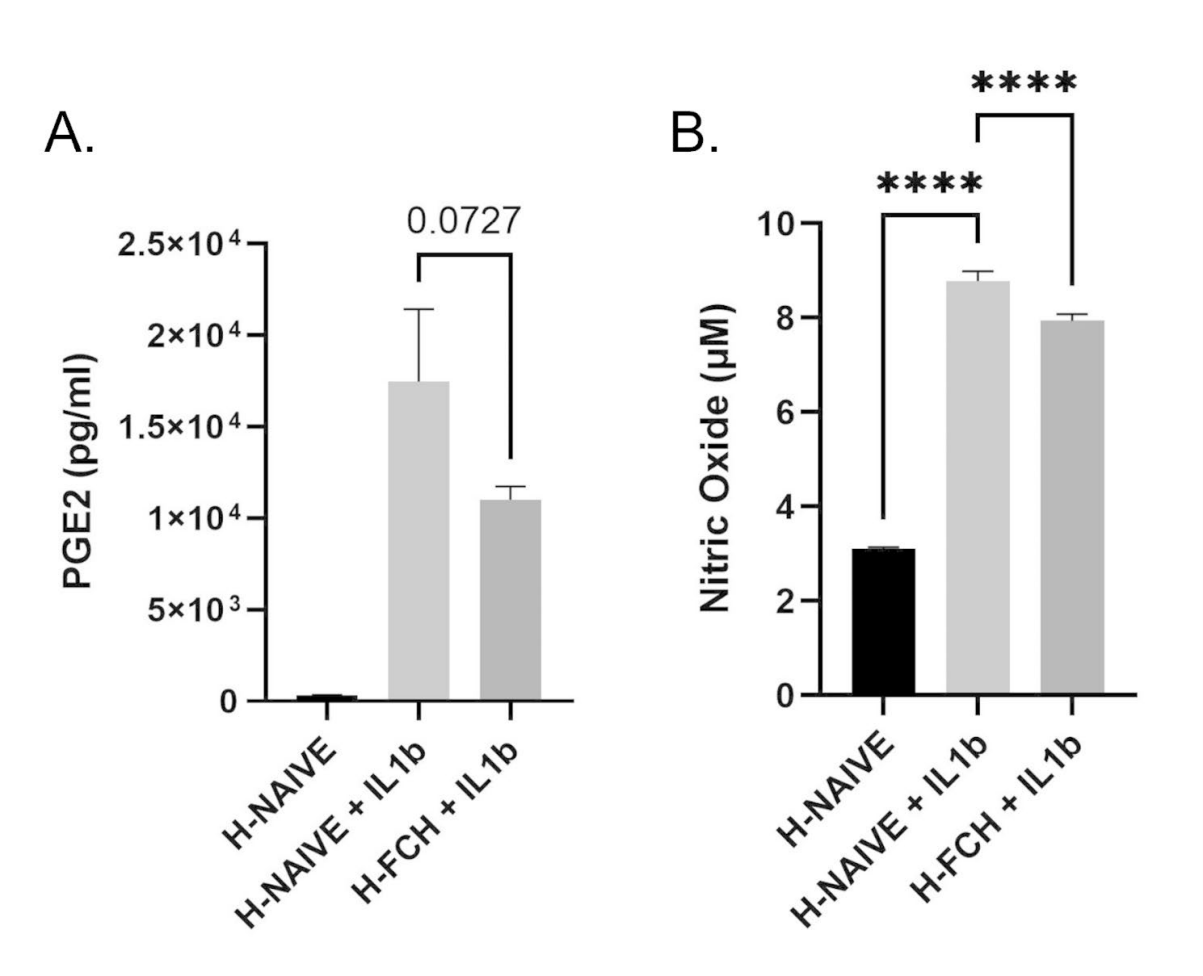
Primary human chondrocytes were incubated in the presence or absence of rhIL-1b (1ng/ml) in combination with either human naive serum (H-NAIVE) or human serum enriched with metabolites derived from FCH ingestion (H-FCH). PGE2 (**A**) and Nitric Oxide (**B**) production. Measures were performed in quadruplicates per condition/volunteer (n = 10 volunteers). Values are presented as mean ± SEM. The differences were considered statistically significant at p < 0.05 with * for p < 0.05; *** for p < 0.001; **** for p < 0.0001

As described in the literature and acknowledged in clinic [36–38], IL-1b promoted the production of both PGE2 and NO by chondrocytes with an up-regulation of + 5284% and + 183% respectively. The presence of FCH metabolites countered both, limiting the rise of PGE2 and NO to + 3298% and + 155% respectively, albeit not significantly in the case of PGE2 (Fig. 7).

## Discussion

In this study, we report the data of an exploratory, non-comparative, multi-centric trial investigating the effect of daily supplementation with FCH on subjects with moderate knee joint discomfort and loss of functionality.

KOOS global score and subscales improved significantly between baseline, 1 month, and 3 months of supplementation, underlining a positive impact of the supplement on joint mobility. Interestingly, an effect was already observed after 1 month of supplementation, which increased with intake duration. While the quality of life of the subjects regarding physical functioning, role limitation due to physical health, energy and fatigue, pain, and general health significantly improved over time, no impact of FCH on the role limitation due to emotional problems, emotional well-being, and social functioning was observed. This could indicate that the effect of FCH on pain is independent of any psychological factors. On the other hand, subjects were selected and thus enriched for physiological parameters such as pain, and results measuring these outcomes are therefore prone to regression to the mean [39], which has been shown to account for up to 60% of the observed change from baseline in symptoms involved in physical exercise studies in OA [40]. One might also argue that the improvement of pain is not sufficient for these subjects to also experience an improvement of the mental components. From a global perspective, the clinical data suggest that FCH improves mobility and decreases pain in subjects suffering from joint discomfort. These observations need however to be interpreted with care due to the exploratory nature of the study, the small sample size, and the absence of a control group. In addition, no radiographic confirmation of joint damage and its level of severity was available, which does not allow any interpretation of the results regarding the effect of FCH on different phenotypes. While this would be interesting, it was not the scope of the present study, which was designed to assess the effect of FCH on a small pilot population of subjects who experience joint discomfort of any kind.

In this context, determination of biological activities of FCH on inflammatory and catabolic pathways may provide further clues. Therefore, to strengthen our conclusions, we designed an *ex vivo* protocol to investigate the influence of FCH circulating human metabolites on primary chondrocyte metabolism. According to the literature, in vitro studies on human or animal chondrocytes suggest an anti-inflammatory and anti-catabolic action of glucosamine and/or chondroitin sulfate [41–44]. Different types of collagen hydrolysate formulations have been shown to stimulate catabolic enzymes such as aggrecanases and matrix metalloproteinases in human cartilage explants. However, these effects were found dependent on the origin of collagen hydrolysate and the authors claimed that the effect of a particular preparation cannot be extrapolated to other formulations [45]. To date, marine collagen hydrolysate has recently been shown to downregulate pro-inflammatory and pro-catabolic markers in chondrocyte organoids in vitro [46, 47]. Here, FCH human metabolites had no effect on aggrecan levels but potently downregulated MMP13 production in rhIL-1β-stimulated primary human chondrocytes. Moreover, we showed that FCH significantly limited the rhIL-1β-related decrease of both collagen II and glycosaminoglycans production, thus further supporting an anti-catabolic role of FCH likely contributing to preserve the mechanical function of cartilage tissue in a context of OA onset.

An anti-inflammatory effect has also been described for eggshell membrane hydrolysate in vitro, another food supplement containing glycosaminoglycans and collagen type I used for the treatment of joint discomfort [48, 49]. In our hands, we demonstrated that FCH metabolites limited the rise of secondary pro-inflammatory mediators by chondrocytes upon rhIL-1β incubation with a trendy but not significant blunt of PGE2 and a very significant reduction of NO production. Interestingly, nitric oxide is a well acknowledged mediator of the nociceptive system in the periphery [50]. Consistently, the reduction of NO may explain, at least partly, the positive influence of FCH on pain as observed in the multicentric clinical trial (exploratory clinical study).

Besides, it is worth noting that even though not statistically significant, a reduction in the use of pain killers could be observed (1 subject took paracetamol after one month and 2 subjects took paracetamol after 3 months, vs. 5 subjects taking paracetamol and 2 subjects taking AINS at baseline). The lack of significance probably stems from the facts that relatively few subjects took pain killers at baseline and any decrease did therefore not have a statistically significant impact due to the overall small sample size. In addition, the safety profile of FCH was found to be very good, which is in line with what has been described for other food supplements [51, 52]. The only serious adverse event reported during the multicentric clinical trial (exploratory clinical study) was unrelated to FCH, and only one third of the declared adverse events were probably or definitively related. Regarding the *ex vivo* clinical trial (*ex vivo* study), no adverse effect was reported. This is of great importance, as standard pharmacological treatments such as analgesics and non-steroidal anti-inflammatory drugs (NSAIDs) can lead to serious side effects such as gastrointestinal bleeding, renal insufficiency, coronary disease, and hepatotoxicity, especially in patients with co-morbidities [7–10, 53]. FCH safety indicates that it could be administrated long-term to prevent and relieve chronic pain in aging patients with comorbidities and that FCH could therefore present a valuable alternative in pain management.

## Conclusions

In conclusion, FCH significantly improved mobility and joint comfort, and reduced pain. It also had a significant impact on the quality of life, highlighted by an improvement of physical health and function. Biological activity of human FCH metabolites give us clues on the mechanisms potentially contributing to the observed articular improvement following FCH supplementation. The observed effects of FCH on mobility and joint comfort will need to be confirmed in a larger randomized placebo-controlled clinical trial, ideally including radiographic or structural changes as well as biochemical markers of joint deterioration. This study will be especially useful for the design of such a randomized placebo-controlled clinical trial, particularly regarding the choice of the primary outcome.

### Electronic supplementary material

Below is the link to the electronic supplementary material.

**Figure S1**. Mean evolution (± SE) of the KOOS sub-scores over time: (a) KOOS Pain score over time; (b) KOOS Symptoms score over time; (c) KOOS Daily living activities score over time; (d) KOOS Sport and recreation function score over time; (e) Quality of life score over time ? FAS population. ***p ≤ 0.001; **p ≤ 0.01; *p ≤ 0.05; ns, not significant. **Figure S2**. Mean evolution (± SE) of the SF-36 sub-scores over time: (a) SF-36 Physical functioning over time; (b) SF-36 Role limitations due to physical health over time; (c) SF-36 Energy/Fatigue over time; (d) SF-36 Pain over time; (e) SF-36 General health over time ? FAS population. ***p ≤ 0.001; **p ≤ 0.01; *p ≤ 0.05; ns, not significant. **Table S1**. Subjects? knee discomfort history description at the inclusion in the study ? FAS population. **Table S2**. Results of the repeated measures ANOVA models for KOOS global score and each subscale, and mean difference and effect size between baseline and 3 months of follow-up ? PP population ? N=24. **Table S3**. Results of the repeated measures ANOVA models for SF-36 global score and each subscale ? PP population ? N=24. **Table S4**. Results of the repeated measures ANOVA models for knee pain at rest and while walking and PGA using VAS scale, and mean difference and effect size between baseline and 3 months of follow-up ? PP population ? N=24. **Table S5**. Comparison of patients? treatment response between the first and the second follow-up ? PP population ? N=24. **Table S6**. Results of the comparison between the first and the second follow-up for compliance? FAS population. **Table S7**. Comparisons of subjects satisfaction between the two follow-up visits ? FAS population. **Table S8**. Results of the analysis of the time evolution for pain killer use and frequency of intake ? FAS population. **Table S9**: Listing of AE and SAE by decreasing order of frequency ? Safety population ? N=28 adverse events. **Table S10**. Distribution of link with FCH and action taken in response to AE ? Safety population ? N=28 adverse events. **Table S11**. Distribution of subjects at each visit ? Safety population ? N=32 patients. **Table S12**. Complete list of exclusion criteria. **Figure S3**. Primary human chondrocytes subjected to ex vivo procedures for validation of cell viability in human serum. Cell viability was measured with an XTT-based assay upon either FCS or human serum incubation (H-NAIVE for human naive serum and H-FCH for human serum enriched with circulating FCH metabolites) for 24 h and 48 h (A and B). Measures were performed in quadruplicates per condition/volunteer (n=10 volunteers). Values are presented as mean ± SD. The differences were considered statistically significant at p < 0.05 with ** for p < 0.01 and **** for p < 0.0001.

## Data Availability

The datasets used and/or analysed during the current study are available from the corresponding author on reasonable request.

## List of abbreviations

AE: Adverse Events
ALT: Alanine aminotransferase
AST: Aspartate aminotransferase
BMI: Body mass index
CS: Chondroitin sulfate
DMB: Dimethylmethylene blue
ES: Effect size
EIA: Enzyme Immunoassay
FCS: Fetal calf serum
FCH: Fish cartilage hydrolysate
FAS: Full analysis set
GGT: Gamma-glutamyltransferase
GS: Glucosamine sulfate
HBSS: Hank’s Balanced sodium Salt
HACs: Human articular chondrocytes
ITT: Intention-to-treat
KOOS: Knee injury and Osteoarthritis Outcome Score
NSAIDs: Nonsteroidal anti-inflammatory drugs
OA: Osteoarthritis
OARSI: Osteoarthritis Research Society International
OMERACT: Outcome Measures in Rheumatoid Arthritis Clinical Trials
PTH: Parathormone
PGA: Patient global assessment
P/S: Penicillin/streptomycin
PP: Per-protocol
QoL: Quality of life
SERM: Selective estrogen-receptor modulator
SAE: Serious Adverse Event
SF-36: Short Form 36 Health Survey
SYSADOA: Symptomatic slow-acting drugs for osteoarthritis
VAS: Visual Analogue Scale
