# Supplementary material for: Oral supplementation with fish cartilage hydrolysate in an adult population suffering from knee pain and function discomfort: results from an innovative approach combining an exploratory clinical study and an ex vivo clinical investigation
Source: BMC Musculoskelet Disord. 2023 Sep 21;24:748. doi: 10.1186/s12891-023-06800-4 (PMC10512646; doi:10.1186/s12891-023-06800-4)
Supplement: Supplementary file 1 — Figure S1. Mean evolution (± SE) of the KOOS sub-scores over time: (a) KOOS Pain score over time; (b) KOOS Symptoms score over time; (c) KOOS Daily living activities score over time; (d) KOOS Sport and recreation function score over time; (e) Quality of life score over time ? FAS population. ***p ≤ 0.001; **p ≤ 0.01; *p ≤ 0.05; ns, not significant. Figure S2. Mean evolution (± SE) of the SF-36 sub-scores over time: (a) SF-36 Physical functioning over time; (b) SF-36 Role limitations due to physical health over time; (c) SF-36 Energy/Fatigue over time; (d) SF-36 Pain over time; (e) SF-36 General health over time ? FAS population. ***p ≤ 0.001; **p ≤ 0.01; *p ≤ 0.05; ns, not significant. Table S1. Subjects? knee discomfort history description at the inclusion in the study ? FAS population. Table S2. Results of the repeated measures ANOVA models for KOOS global score and each subscale, and mean difference and effect size between baseline and 3 months of follow-up ? PP population ? N=24. Table S3. Results of the repeated measures ANOVA models for SF-36 global score and each subscale ? PP population ? N=24. Table S4. Results of the repeated measures ANOVA models for knee pain at rest and while walking and PGA using VAS scale, and mean difference and effect size between baseline and 3 months of follow-up ? PP population ? N=24. Table S5. Comparison of patients? treatment response between the first and the second follow-up ? PP population ? N=24. Table S6. Results of the comparison between the first and the second follow-up for compliance? FAS population. Table S7. Comparisons of subjects satisfaction between the two follow-up visits ? FAS population. Table S8. Results of the analysis of the time evolution for pain killer use and frequency of intake ? FAS population. Table S9: Listing of AE and SAE by decreasing order of frequency ? Safety population ? N=28 adverse events. Table S10. Distribution of link with FCH and action taken in response to AE ? Safety population ? N=2 [file 12891_2023_6800_MOESM1_ESM.docx]

**Supplemental Material**
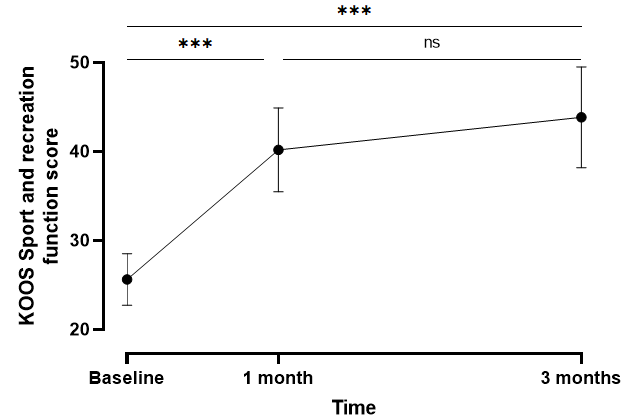
**
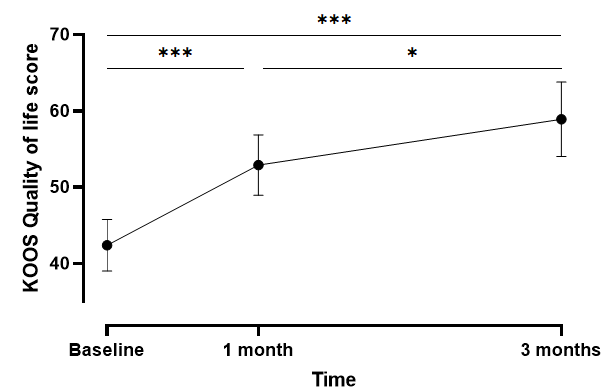

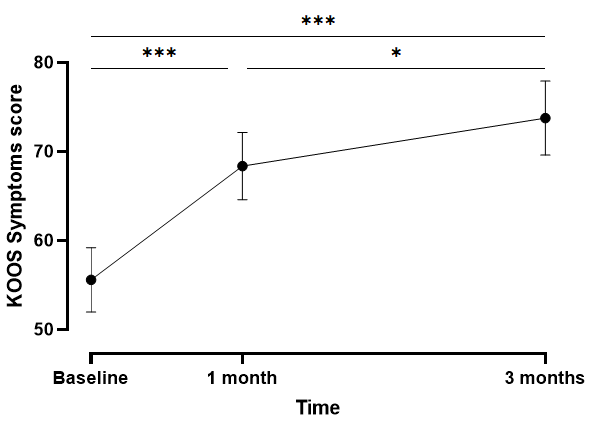

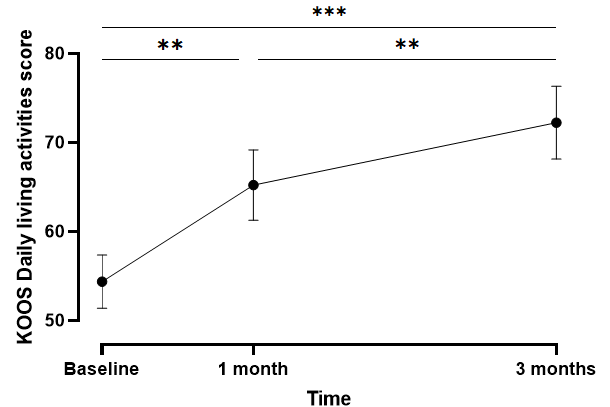

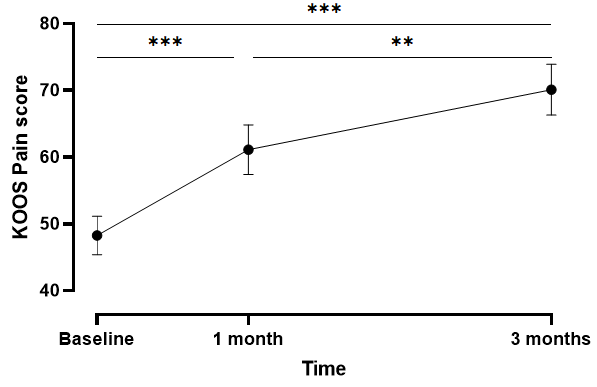
**

**

*

***

***

ns

***

**

**

***

(a)

(b)

(c)

(d)

(e)

(a)

(b)

(c)

(d)

(e)

**Figure S1.** Mean evolution (± SE) of the KOOS sub-scores over time: (a) KOOS Pain score over time; (b) KOOS Symptoms score over time; (c) KOOS Daily living activities score over time; (d) KOOS Sport and recreation function score over time; (e) Quality of life score over time – FAS population. ***p ≤ 0.001; **p ≤ 0.01; *p ≤ 0.05; ns, not significant.

**
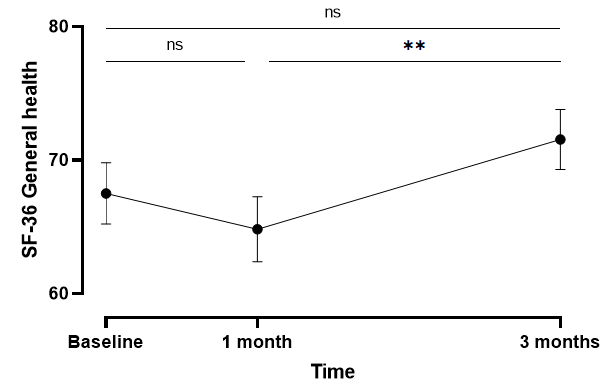

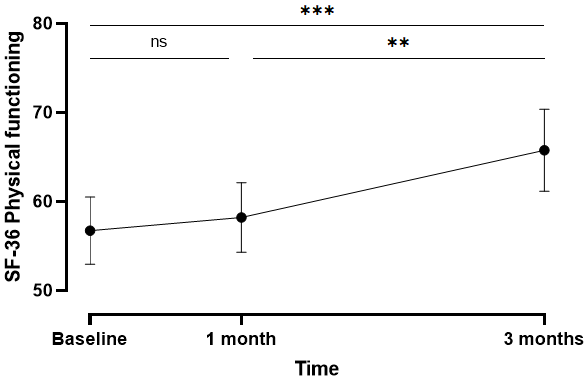

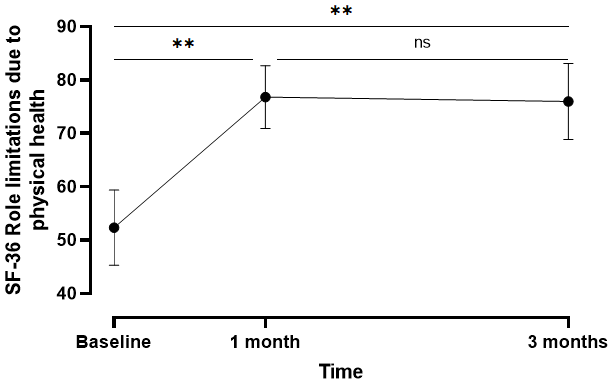

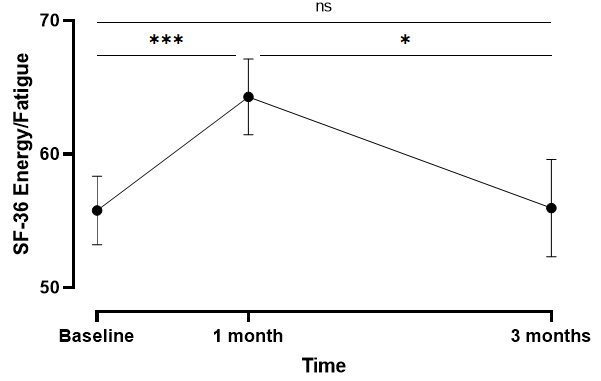

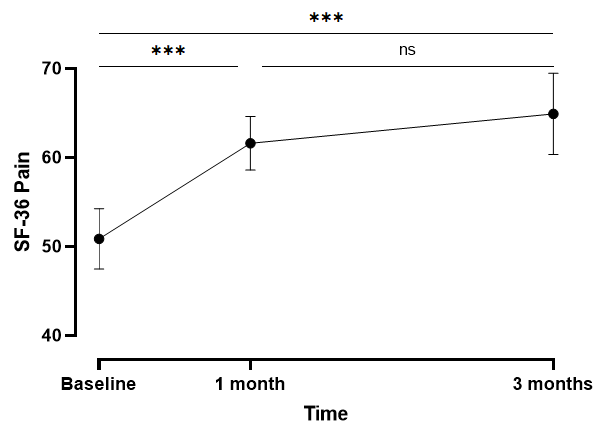
s**

(a)

(b)

(c)

(d)

(e)

**Figure S2.** Mean evolution (± SE) of the SF-36 sub-scores over time: (a) SF-36 Physical functioning over time; (b) SF-36 Role limitations due to physical health over time; (c) SF-36 Energy/Fatigue over time; (d) SF-36 Pain over time; (e) SF-36 General health over time – FAS population. ***p ≤ 0.001; **p ≤ 0.01; *p ≤ 0.05; ns, not significant.

| **Table S1.** Subjects’ knee discomfort history description at the inclusion in the study – FAS population | | | | | | | | | | |
| --- | --- | --- | --- | --- | --- | --- | --- | --- | --- | --- |
| Variable | N | Category | N(%) | Mean | SD | Min | Q1 | Median | Q3 | Max |
| Knee | 32 |  |  |  |  |  |  |  |  |  |
|  |  | Left | 15 (46.9) |  |  |  |  |  |  |  |
|  |  | Right | 17 (53.1) |  |  |  |  |  |  |  |
| VAS at rest (mm) | 32 |  |  | 53.66 | 11.95 | 40.00 | 43.00 | 50.00 | 60.50 | 89.00 |
| VAS while walking (mm) | 32 |  |  | 61.56 | 21.30 | 14.00 | 50.00 | 60.00 | 75.00 | 100.00 |
| VAS for PGA (mm) | 32 |  |  | 64.28 | 12.90 | 40.00 | 55.00 | 64.00 | 70.50 | 99.00 |

|  | **Table S2.** Results of the repeated measures ANOVA models for KOOS global score and each subscale, and mean difference and effect size between baseline and 3 months of follow-up – PP population – N=24 | | | | | | |
| --- | --- | --- | --- | --- | --- | --- | --- |
| Variable | | Baseline (n=24) | After 1 month (n=24) | After 3 months (n=24) | P-value | Mean difference (n=24) | Effect size |
| Global score [mean±SD] | | 42.28±13.45 | 55.30±18.18 | 62.42±20.21 | <.0001 | 20.14±16.42 | 1.23 |
| Pain* [mean±SD] | | 45.49±14.76 | 59.84±18.71 | 68.75±19.23 | <.0001 | 23.26±18.39 | 1.26 |
| Symptom [mean±SD] | | 53.27±20.77 | 66.52±20.23 | 72.92±21.43 | <.0001 | 19.64±20.72 | 0.95 |
| Activity of daily living [mean±SD] | | 52.33±17.52 | 62.75±20.80 | 70.53±20.68 | <.0001 | 18.20±13.76 | 1.32 |
| Sport and recreation function [mean±SD] | | 23.33±15.86 | 36.88±24.40 | 42.08±27.50 | 0.0001 | 18.75±23.23 | 0.81 |
| Quality of life* [mean±SD] | | 26.98±14.96 | 50.52±19.15 | 57.81±24.33 | <.0001 | 20.83±18.86 | 1.10 |
| *log-transformed variable | | | | | |  |  |

| **Table S3.** Results of the repeated measures ANOVA models for SF-36 global score and each subscale – PP population – N=24 | | | | |
| --- | --- | --- | --- | --- |
| Variable | Baseline (n=24) | After 1 month (n=24) | After 3 months (n=24) | P-value |
| Global score [mean±SD] | 56.84±15.51 | 63.76±13.01 | 65.30±15.85 | *0.0045* |
| Physical functioning [mean±SD] | 51.88±21.36 | 54.17±19.09 | 63.96±23.50 | *0.0011* |
| Role limitations due to physical health [mean±SD] | 45.83±38.78 | 72.92±32.06 | 73.96±37.21 | *0.0025* |
| Role limitation due to emotional problems [median (Q1-Q3)] | 100.00 (50.00-100.00) | 100.00 (66.67-100.00) | 100.00 (33.33-100.00) | 0.9406* |
| Energy/fatigue [mean±SD] | 51.67±13.24 | 62.29±14.59 | 54.58±18.53 | *0.0092* |
| Emotional well-being [mean±SD] | 60.83±19.31 | 66.00±17.46 | 60.67±18.45 | 0.1491 |
| Social functioning [median (Q1-Q3)] | 75.00 (56.25-87.50) | 75.00 (62.50-87.50) | 75.00 (62.50-93.75) | 0.7066* |
| Pain [mean±SD] | 44.79±14.35 | 58.65±14.29 | 61.98±21.83 | *<.0001* |
| General health [mean±SD] | 67.71±12.77 | 65.21±11.75 | 70.83±11.58 | 0.0585 |
| * Friedman’s test p-value | | | | |

|  | **Table S4**. Results of the repeated measures ANOVA models for knee pain at rest and while walking and PGA using VAS scale, and mean difference and effect size between baseline and 3 months of follow-up – PP population – N=24 | | | | | | |
| --- | --- | --- | --- | --- | --- | --- | --- |
| Variable | | Baseline (n=24) | After 1 month (n=24) | After 3 months (n=24) | P-value | Mean difference (n=24) | Effect size |
| At rest (mm)* [mean±SD] | | 53.29±12.40 | 36.88±19.51 | 23.00±19.26 | *0.0007* | -30.29±21.83 | 1.39 |
| While walking (mm) [mean±SD] | | 63.17±20.07 | 45.46±21.42 | 34.04±21.22 | *<.0001* | -29.13±30.85 | 0.94 |
| PGA (mm) [mean±SD] | | 65.42±13.75 | 40.29±21.12 | 32.75±24.91 | *<.0001* | -32.67±32.21 | 1.01 |
| *log-transformed variable | | | | | |  |  |

| **Table S5.** Comparison of patients’ treatment response between the first and the second follow-up – PP population – N=24 | | | | | |
| --- | --- | --- | --- | --- | --- |
|  |  | After 3 months [N (%)] | | |  |
|  |  | No | Yes | Total | P-value |
| After 1 month [N (%)] | No | 6 (25.0) | 6 (25.0) | 12 (50.0) | 0.1573 |
|  | Yes | 2 (8.3) | 10 (41.7) | 12 (50.0) |  |
|  | Total | 8 (3.3) | 16 (6.7) |  |  |

| **Table S6.** Results of the comparison between the first and the second follow-up for compliance– FAS population | | | | |
| --- | --- | --- | --- | --- |
| Variable | After 1 month (n=19) | After 3 months (n=16) | Difference (n=11) | P-value |
| Compliance (%) [median (Q1-Q3)] | 100.00 (97.65-100.00) | 99.14 (95.24-100.00) | 0.00 (-6.70-2.78) | 0.4922 |

| **Table S7.** Comparisons of subjects satisfaction between the two follow-up visits – FAS population | | | | |
| --- | --- | --- | --- | --- |
| Variable | After 1 month (n=26) | After 3 months (n=26) | Difference (n=26) | P-value |
| Odour [median (Q1-Q3)] | 3.0 (2.0-4.0) | 3.0 (2.0-4.0) | 0.0 (-0.5-0.0) | 0.6289 |
| Taste [median (Q1-Q3)] | 4.0 (3.0-4.0) | 4.0 (3.0-4.0) | 0.0 (-0.5-1.0) | 0.8167 |
| Oral intake [median (Q1-Q3)] | 4.0 (3.0-4.0) | 4.0 (4.0-4.0) | 0.0 (0.0-1.0) | 0.1826 |
| Digestion [median (Q1-Q3)] | 4.0 (3.0-4.0) | 4.0 (4.0-4.0) | 0.0 (0.0-0.5) | 0.5898 |
| Effects on symptoms or on quality of life [median (Q1-Q3)] | 3.5 (3.0-4.0) | 4.0 (3.0-4.0) | 0.0 (-0.5-1.0) | 0.7032 |

| **Table S8.** Results of the analysis of the time evolution for pain killer use and frequency of intake – FAS population | | | | | |
| --- | --- | --- | --- | --- | --- |
| Pain Killer | Baseline | After 1 month | After 3 months | N (%) | P-value |
| **Use of pain killer** | | | |  |  |
| Paracetamol | No | No | No | 19 (73.1) | 0.2462 |
|  | Yes | No | No | 4 (15.4) |  |
|  | No | No | Yes | 2 (7.7) |  |
|  | Yes | Yes | No | 1 (3.8) |  |
| AINS | No | No | No | 24 (92.3) | - |
|  | Yes | No | No | 2 (7.7) |  |
| **Frequency of pain killer intake** [median (Q1-Q3)] | | | | | |
| Paracetamol | 0.0 (0.0-0.0) | 0.0 (0.0-0.0) | 0.0 (0.0-0.0) |  | 0.1561 |
| AINS | 0.0 (0.0-0.0) | 0.0 (0.0-0.0) | 0.0 (0.0-0.0) |  | 0.1353 |

| **Table S9** : Listing of AE and SAE by decreasing order of frequency – Safety population – N=28 adverse events | |
| --- | --- |
| MedDRA name | N(%) |
| All adverse events | |
| Headache | 4 (14.3) |
| Constipation | 2 (7.1) |
| Ageusia | 1 (3.6) |
| Attack heart (NOS) | 1 (3.6) |
| Blood pressure fluctuation | 1 (3.6) |
| Diarrhea | 1 (3.6) |
| Emotional instability | 1 (3.6) |
| External compression headache | 1 (3.6) |
| Flu like symptoms | 1 (3.6) |
| Gastralgia | 1 (3.6) |
| Hand Pain | 1 (3.6) |
| Heartburn | 1 (3.6) |
| Knee derangement | 1 (3.6) |
| Loose stools | 1 (3.6) |
| Low back pain | 1 (3.6) |
| Lumbago | 1 (3.6) |
| Muscle pain | 1 (3.6) |
| Paresis | 1 (3.6) |
| Skin rash | 1 (3.6) |
| Tingling feet/hands | 1 (3.6) |
| Tiredness | 1 (3.6) |
| Urinary tract infection | 1 (3.6) |
| Vomiting | 1 (3.6) |
| Weight gain | 1 (3.6) |
| Serious adverse events | |
| Attack heart (NOS) | 1 (100.0) |

| **Table S10.** Distribution of link with FCH and action taken in response to AE – Safety population – N=28 adverse events | | |
| --- | --- | --- |
| Link with FCH | Action taken in response to AE | N(%) |
| All adverse event | | |
| Unlikely | None | 17 (60.7) |
| Unlikely | Suspension | 1 (3.6) |
| Probable | None | 3 (10.7) |
| Probable | Suspension | 2 (7.1) |
| Related | None | 2 (7.1) |
| Related | Definitive withdrawal | 2 (7.1) |
| Unrelated | Definitive withdrawal | 1 (3.6) |
| Serious adverse event | | |
| Unrelated | Definitive withdrawal | 1 (100.0) |

| **Table S11.** Distribution of subjects at each visit – Safety population – N=32 patients | | |
| --- | --- | --- |
|  | Complete visit: N (%) | Incomplete visit: N (%) |
| At baseline | 32 (100.0) | 0 (0.0) |
| After 1 month | 28 (87.5) | 4 (12.5) |
| After 3 months | 26 (81.3) | 6 (18.7) |

| **Table S12**. Complete list of exclusion criteria |
| --- |
| **Related to knee** |
| •        Recent trauma (< 1 month) of the knee responsible for the symptomatic knee; |
| •        Concurrent articular disease interfering with the evaluation of knee pain left to the Investigator’s discretion; |
| •        Prosthesis in the target knee. |
| **Related to treatments** |
| •        Analgesics to manage knee pain 24 hours before inclusion visit; |
| •        Corticosteroid injection in the target knee in the last month; |
| •        Hyaluronan injection in the target knee in the last 6 months; |
| •        Arthroscopy in the last 6 months; |
| •        Oral corticotherapy ≥ 5mg/day (in Prednisolone equivalent) in the last 3 months; |
| •        Symptomatic slow-acting drugs for osteoarthritis (SYSADOA) or dietary supplement, i.e., Curcuma extract, chondroitin, glucosamine, diacerein, or avocado-soya unsaponifiables in the last 3 months; |
| •        Contraindications to CARTIDYSS^®^: hypersensitivity or allergy to the product components (fish); |
| •        Treatments based on strontium ranelate, bisphosphonates, selective estrogen-receptor modulator (SERM), and parathormone (PTH) in the last 12 months; |
| **Related to associated diseases** |
| •        Any severe, uncontrolled, and limiting diseases left to the Investigator’s discretion; |
| •        Anticipated need for any surgical or other invasive procedure during the trial including prosthesis in the target knee; |
| •        Anticipated need for any forbidden treatments during the trial; |
| •        Swallowing disorder; |
| •        Patient with widespread pain/depression (e.g., fibromyalgia). |
| **Related to patients** |
| •        Close collaborators to the investigational team, the study coordinator (Artialis) or to the Sponsor (Abyss Ingrédients); |
| •        Currently participating or having participated in another therapeutic clinical trial in the last 3 months; |
| •        Under guardianship or judicial protection; |
| •        Pregnancy, breastfeeding, planned conception, or premenopausal women without effective contraception (pill, patch, ring, diaphragm, implant, and intrauterine device), tubal ligation, or hysterectomy. |


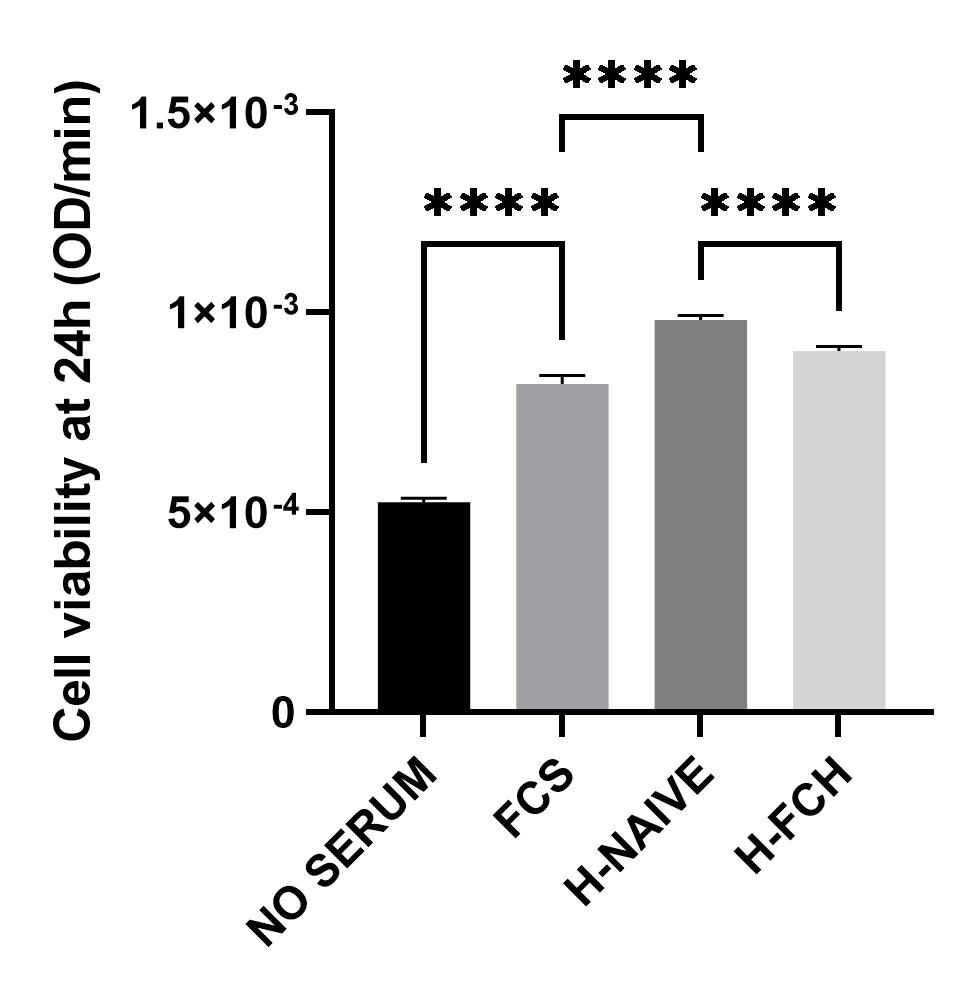

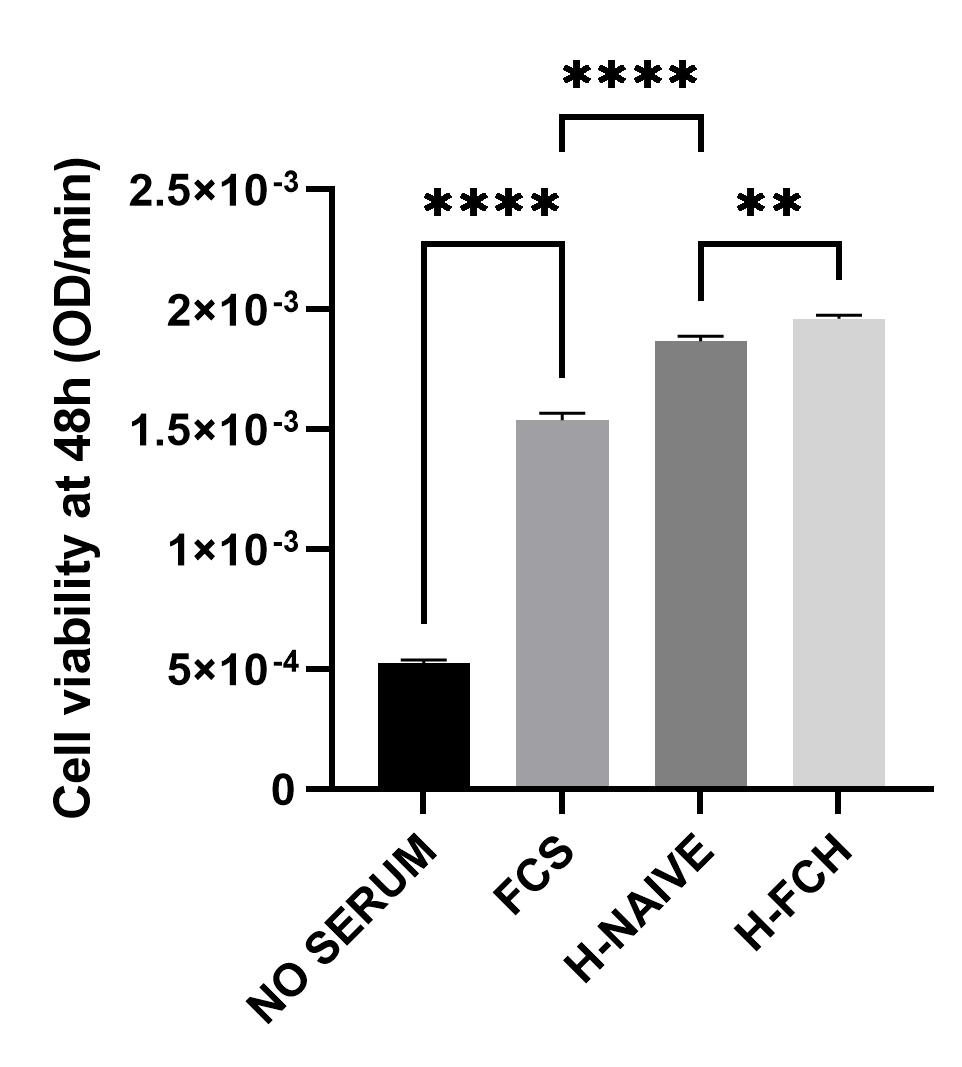


B.

A.

**Figure S3.** Primary human chondrocytes subjected to *ex vivo* procedures for validation of cell viability in human serum. Cell viability was measured with an XTT-based assay upon either FCS or human serum incubation (H-NAIVE for human naive serum and H-FCH for human serum enriched with circulating FCH metabolites) for 24 h and 48 h (A and B). Measures were performed in quadruplicates per condition/volunteer (n=10 volunteers). Values are presented as mean ± SD. The differences were considered statistically significant at p< 0.05 with ** for p< 0.01 and **** for p< 0.0001.
